# Conspiracy theories as quasi-religious mentality: an integrated account from cognitive science, social representations theory, and frame theory

**DOI:** 10.3389/fpsyg.2013.00424

**Published:** 2013-07-16

**Authors:** Bradley Franks, Adrian Bangerter, Martin W. Bauer

**Affiliations:** ^1^Institute of Social Psychology, London School of Economics and Political ScienceLondon, UK; ^2^Institute of Work and Organizational Psychology, University of NeuchâtelNeuchâtel, Switzerland

**Keywords:** quasi-religion, social representations, frame theory, conspiracy theory, minimal counter-intuitiveness, sense making, beliefs

## Abstract

Conspiracy theories (CTs) can take many forms and vary widely in popularity, the intensity with which they are believed and their effects on individual and collective behavior. An integrated account of CTs thus needs to explain how they come to appeal to potential believers, how they spread from one person to the next via communication, and how they motivate collective action. We summarize these aspects under the labels of *stick*, *spread*, and *action*. We propose the quasi-religious hypothesis for CTs: drawing on cognitive science of religion, social representations theory, and frame theory. We use cognitive science of religion to describe the main features of the content of CTs that explain how they come to stick: CTs are quasi-religious representations in that their contents, forms and functions parallel those found in beliefs of institutionalized religions. However, CTs are *quasi-religious* in that CTs and the communities that support them, lack many of the institutional features of organized religions. We use social representations theory to explain how CTs spread as devices for making sense of sudden events that threaten existing worldviews. CTs allow laypersons to interpret such events by relating them to common sense, thereby defusing some of the anxiety that those events generate. We use frame theory to explain how some, but not all CTs mobilize collective counter-conspiratorial action by identifying a target and by proposing credible and concrete rationales for action. We specify our integrated account in 13 propositions.

## INTRODUCTION

Conspiracies are a fact of history, so much that Machiavelli (1532/1961) wrote a famous practical guide on how to conspire successfully against an illegitimate government. However, conspiracy theories (CTs) are often independent from the base rate of real conspiracies (Bale, 2007). CTs may over-generalize a cognitive and social possibility out of proportion, seeing agency where there may be little more than randomness, “cock-up” or structural dynamics which nobody in particular designed.

However, our present purpose is not to debunk yet another CT, but to theoretically elucidate their dynamics and inner logic. Like Freud (1914) considered slips of the tongue as openings to the unconscious mind, Norman (1981) presented action slips as markers of the organization of memory, and Reason (1990) uses errors to gain insights into skilled performance, we consider CTs as occasions to elucidate actual representations of reality as part of human social functioning. Understanding CTs in turn helps us understand the potentially high human costs of over-generalizing agency. Though we need to guard ourselves against creating a conspiracy theory of CTs, our working hypothesis is that CTs have a degree of functional autonomy in modern societies which needs to be understood.

Conspiracy theories have many facets. On the one hand, they constitute cognitive resources that fulfill a need to explain unusual, disturbing events such as disease outbreak, disruptive technology, major scandal, or sudden celebrity death (McCauley and Jacques, 1979; Wagner-Egger et al., 2011). On the other hand, they are narratives that circulate in culture – in mass media, as rumors, in stories (Byford, 2011). These narratives reduce the complexity presented by such events, contain the uncertainty they generate, and translate unspecific anxiety into focused fears (Barrett and Lawson, 2001). CT narratives are also inscribed in the context of antagonistic relations between groups, drawing on recurrent negative views of outgroups to explain events and, sometimes, motivate collective action.

The interplay between psychological and socio-cultural functions of CTs is arguably best understood in interdisciplinary terms. CTs have attracted research in pockets of social science and humanities like cultural studies (Parish and Parker, 2001), psychoanalysis (Zonis and Joseph, 1994), political science (Hofstadter, 1965), history (Roberts, 1972), philosophy (Keeley, 1999), and psychology (Swami et al., 2011). Whilst insightful, such research has investigated CTs from within a discipline, focusing either on symbolic aspects (e.g., rhetoric, discourse, narratives; Billig, 1987) or on psychological phenomena (cognitive biases and errors, individual differences, psychopathology), but there have been few attempts at integration – leaving a more general theory of CTs lacking.

It is difficult to explain the set of dimensions along which CTs vary from within specific disciplines. One dimension concerns the intensity of belief in the CT, which ranges from casual to passionate. More casual CTs (e.g., the “X-Files”), can be engaged with similar to any kind of fiction, suspending disbelief whilst being entertained (Sunstein and Vermeule, 2009). But other CTs are believed quite passionately, and are used to frame events in radical ways so as to mobilize collective action against a target (the conspiratorial agent). An example is the CT propagated during the Black Death accusing Jews of plotting against Christianity. This CT motivated Christian inhabitants of European cities to numerous pogroms, leading to the eradication of Jewish communities in Western Europe (Kelly, 2005). A second dimension of CTs is their distribution. Some CTs arise in a small community and are not widely accepted in society (e.g., the belief that powerful leaders of this planet are members of a race of lizards seeking world domination), while others are held by a majority of the population (e.g., the belief that the Kennedy assassination was not the work of a lone gunman; Goertzel, 1994). A third dimension along which CTs vary is their connection with collective action – some being confined to collective sense-making, whilst others (as in the pogroms) lead to collective action against the alleged conspirators.

The variety of CTs along the three dimensions of belief intensity, distribution and action (more could be distinguished) thus poses a challenge to any erstwhile account of this genre. We summarize these aspects under the labels of *stick*, *spread*, and *action*, and propose an account of CTs that explains them by drawing on three theoretical approaches: the cognitive science of religion, social representations theory, and frame theory.

We propose the quasi-religious hypothesis for CTs: CTs are quasi-religious representations, in that their contents, forms, and functions parallel those found in beliefs supported by institutionalized religions, though CTs lack certain features of organized religions. Being quasi-religious offers an explanation of CTs’ ubiquity, especially in postindustrial secular societies. But CTs appeal especially to constituencies who are averse to the strictures of organized religions or established political orthodoxies. CTs have a subversive flavor that contradicts official accounts of events, be they secular or religious. This feature is difficult to explain via cognitive science, but is a primary focus of social representations theory. Social representations theory explains how CTs enable laypersons to make sense of complex, ambiguous situations, how CTs spread, and how they may change during spreading. However, social representations theory does not focus primarily on how representations get used by social groups to achieve political ends, and so we invoke frame theory to deal with this aspect.

We review relevant aspects of cognitive science of religion, social representations theory, and frame theory, applying them to the analysis of CT features. This generates a set of propositions about CTs that can be used to develop further empirical investigations. In what follows, we use specific terminology: CTs are propagated by *sponsors* who seek to *spread* *sticky* representations of events to a larger audience, often with the intent to *frame* them into action.

## EXPLAINING CT STICKINESS: COGNITIVE SCIENCE OF RELIGION

Our key claim is that CTs have contents and functions that are quasi-religious: there is an *analogy* between religious beliefs and some of the ways in which CTs typically construe the issue and the conspirators. There is, however, no predicted *homology* between CTs and religious beliefs – they are not identical in content, structure or function, nor is there any predicted relation between religiosity – the tendency to hold religious beliefs – and the tendency to hold CTs. Indeed, the value of a scientific analogy is partly in the ways in which the two domains match and partly in the way in which they do not; as we note below, both may be empirically informative.

### RELIGION, COGNITIVE SCIENCE, AND ANTHROPOLOGY

Cognitive science and cognitive anthropology of religion treat supernatural beliefs as natural, in that they have the same foundation as beliefs about the natural and social world (e.g., Boyer, 2001; Boyer and Ramble, 2001; see also Pyysiäinen, 2001; Atran and Norenzayan, 2004). However, instead of simply instantiating all intuitive beliefs, religious beliefs are “minimally counter-intuitive,” in denying or violating a few intuitive qualities – for example, a representation of a “spirit” is founded on commonsense representations about people (e.g., people have agency, act according to beliefs and desires, have memories), but denies qualities concerning their physical and biological nature. This minimal biological counter-intuitiveness then enables the belief in further qualities of spirits (e.g., their longevity, ability to pass through physical objects). By building on everyday representations of the natural and social world, religious representations therefore draw on explanatory resources used in everyday life: the supernatural builds on the natural.

Three additional qualities of religious representations are pertinent to CTs. How those representations are held in mind (i.e., *how* they are believed), the specific contents that are represented (i.e., *what* is believed), and *why* other peoples’ beliefs should matter to us. Concerning how they are believed, Sperber (1975, 1996) has argued that some religious representations involve meta-representations: paradoxical representations that are not fully interpreted, in comparison to the non-religious representations on which they are based – there is always a kernel of doubt at their core, arising from their empirical indeterminacy (Franks, 2003). For example, Catholic mass involves ingesting a solid and a liquid that are simultaneously bread and wine and the body and the blood of Christ. Whereas representations of bread, wine, the body and the blood of Christ are easily understood as solids and liquids, the meta-representation of the two solids as identical, and the two liquids as identical, remains a mystery. Sperber (1975, 1996) suggests that the identification is held in mental quotes, and is not fully processed. Adherents are not expected to fully understand such a representation to the degree that they would understand ordinary representations; rather, they defer to the expert knowledge of priests. Complex, mysterious and potentially threatening ideas can be explained, if not by the adherents themselves. Deference to experts is, therefore, often built into religious beliefs.

Regarding content of religious beliefs, representations of supernatural agents often involve denying human physical qualities but retaining or exaggerating psychological ones. In particular, they tend to interpret situations or events via the activity of sentient agents rather than situational factors. This may reflect an evolved disposition to see agency where there is none (Guthrie, 1980, 1993); such “hyperactive” agency detection would lead people to make more false positive judgements than false negative judgements regarding whether, for example, a surprising sound like a snapping twig was caused by a predator or a harmless event. It is a small step from this to explaining complex events that generate ontological anxieties (illness, crop failure, unexpected death of loved ones) by the intentional actions of a minimally counter-intuitive agent – a supernatural agent. Selective pressure associated with “hyperactive” agency detection might explain the prevalence of counter-intuitive religious concepts that involve supernatural agency relative to those that do not. Such concepts express the abdication of an individual’s own agency (or an awareness of one’s own lack of agency) regarding ontologically important events and activities.

Religions also ascribe particular psychological qualities to supernatural agents (Boyer, 2001). Put simply, gods punish and benefit believers according to the degree of their adherence to doctrine (see also Barrett, 2004). They are not understood as omniscient or as “full access agents” regarding adherents’ mental states and experiences, irrespective of the prescriptions of formal theology (Barrett and Keil, 1996). Instead, they are “strategic access agents”: they are omniscient regarding *only* adherents’ beliefs, desires, and intentions regarding morally relevant actions that affect their group. Group members follow the god’s prescriptions for punishing dissent and praising assent, in the service of sacred values (Atran, 2002). Being a member of such a group thereby involves the minimally counter-intuitive representation of a supernatural agent who polices the group’s moral boundaries, supporting co-operation, altruism and other prosocial behavior regarding the ingroup. This explains why other peoples’ religious beliefs should matter to us – they justify sharing resources with other believers, withholding resources from non-believers, punishing defectors, and the more general intra-group and inter-group dynamics widely studied in social psychology. If outgroups or events challenge the ingroup’s sacred values, it experiences an intense sense of threat since these are precisely the values that ground ingroup identity and cement its connection to the deity. The more inter-group conflict affects sacred values, the more intractable that conflict becomes (Atran and Ginges, 2012).

Demarcation along religious lines facilitates thinking about groups in essentialist terms (e.g., Hirschfeld, 1996; Gelman and Hirschfeld, 1999; Haslam et al., 2000; Gil-White, 2001; Gelman, 2005). Religious groups often construe members as “fictive kin” (e.g., fellow believers as “brothers” or “brethren”). This supports extending altruism to fictive kin, treating threats to fictive kin as if they were threats to genetic kin, and supports the essentialization of religious group demarcations. Essentialism suggests that beneath surface qualities there are deep qualities that confer group membership. People may represent empty “place holders” for the essences: they believe there is an essence without knowing precisely what it is. As with the “quoted” representations discussed above, adherents may defer to authority to obtain information about the essence or to obtain “permission” to cease searching for it (see Medin and Ortony, 1989). Essentialized group differences are therefore resistant to counter-examples: they can be accommodated because the representation is just a place holder. The qualities that “fill” the place holder can change, thus preserving the essentialist structure of explaining surface differences by deep differences.

Counter-intuitive representations get stickier by being embedded in broader narratives. Norenzayan et al. (2006) found that narratives including mostly intuitive representations, but one or two minimally counter-intuitive representations, were more likely to be recalled after a delay than narratives comprising wholly intuitive representations or ones which were maximally counter-intuitive. Such a structure is also found in culturally stable narratives like fairy tales. It is thus the interplay of intuition and counter-intuition, natural and supernatural, that makes religious representations cognitively attractive, and contributes to their stickiness (see also Barrett and Nyhof, 2001).

But religions are not only concerned with essentialized groups and supernatural agents. They also involve rituals; indeed, believed representations and rituals are mutually reinforcing (e.g., McCauley and Lawson, 2002: Whitehouse, 2004). Ritual provides affective or emotional validation for beliefs not otherwise amenable to empirical verification. Performing the ritual provides adherents with the conviction that those beliefs are validated (Franks, 2004; Bloch, 2005). Religious rituals vary in their nature (Whitehouse, 2004), from the more intensely emotional and “imagistic” (like baptism or funeral) to the more “intellectual” and “doctrinal” (like repeating catechism). Religious rituals manage ontological anxiety in daily life via social activities performed by ingroup members, drawing on specific intra-group and inter-group hopes and fears, threats and other dynamics (Atran, 2002). Moreover, repetition embeds the ritual in the group’s norms, making it more central to the group’s beliefs (Barrett and Lawson, 2001). In this way, ritual acts signal historical continuity of group membership, linking current adherents to those of the past and recapitulating past inter-group relations. By performing rituals accurately, and by engaging in appropriate intra-group co-operation and punishment and inter-group competition and derogation, diffuse ontological anxieties are translated into concrete fears managed by particular actions.

Other insights on how religious beliefs vary come from sociology of religion. One way they vary (besides specific contents of the beliefs), is in their enactment – the extent of involvement in activities beyond private belief. In economically developed nations, a functional, “secular” religiosity has emerged as a redirected focus for religious thinking (Luckmann, 1990) where many people have ceased to adhere to traditional religions. Such “New Age” beliefs express a form of religious “bricolage” or “do it yourself,” where the individual selects congenial elements from different religions (Luckmann, 1979). Berger (1967) and Beyer (1990) refers to this as a privatization of religion, “repackaged” for individual consumption. Such a pattern may dilute the intrinsic connection between religious belief and shared ritual mentioned above. In a related vein, Stark and Bainbridge (1987) noted three types of activity, which they call “cult” involvement. An audience cult is**the most private form of adherence, and involves interest in religion, but perhaps only reading the occasional book or listening to the occasional lecture. A client cult involves contact with other practitioners, but may only involve learning specific techniques (e.g., completing a meditation course) as opposed to an ongoing commitment. Finally, a cult movement involves conversion accompanied by regular involvement in a religious lifestyle. Of course, even this is not the most intense form of commitment. Still others may go beyond “cult movement” to adopt specific perspectives on the religion, e.g., by special additional training, ritual involvement, and authority sanctioning to become a privileged member of the group (e.g., a priest).

A further step in religious involvement is engaging in fundamentalist beliefs and associated practices. The religious ingroup is seen as engaged in a battle in response to a crisis – a cosmic war between forces of good and evil. According to Marty and Appleby (1994, 2004), fundamentalism involves a sense of conflict with “enemies” from within, who departed from the original path, and from without, whose secularist beliefs and policies are inimical to religion itself, so that the religion is in danger of annihilation.

### APPLICATION TO CTs

Our suggestion is that CTs are analogous to religious beliefs. There may be variations in how each CT expresses quasi-religiousness. Our suggestion is *not* that CTs possess more or fewer quasi-religious qualities, but that all significant CTs possess quasi-religious qualities, each to varying degrees. That is, we are not claiming that some CTs have minimally counter-intuitive representations, and others do not; rather, we claim that they all do, but that they vary in complexity and depth of such qualities. An “ideal type” of a CT may possess all of the quasi-religious qualities to a significant degree of complexity and depth, whilst specific instances diverge in different ways. Any dis-analogy may itself be informative – just as we can ask why some CTs do not engender strong social movements, we might also ask why some CTs generate the fervor of strongly held religious beliefs, whilst others do not. We now articulate the main aspects of this ideal type as a set of empirically tractable propositions.

Our overarching suggestion is that CTs draw on resources employed by religious representations. CTs thus merge the intuitive and the counter-intuitive in response to challenging secular events. Events are construed as resulting from the actions of an outgroup that threatens values sacred to ingroup. This leads us to Proposition 1 with three components.

#### Proposition 1: CT success or “stickiness” is a function of minimal counter-intuitiveness

***Proposition 1a: conspiring agents in successful CTs are represented as minimally counter-intuitive in strategic omniscience and strategic omnipotence.*** Characterizing the conspiring agent as possessing minimally counter-intuitive powers and knowledge seems a core quality of CTs (Shermer, 2012). The agent is rarely outlandishly or bizarrely powerful or strange, except in extreme cases. Such cases function as rhetorical devices to deliberately contrast with, and so render more credible, the less bizarre qualities of agents in other CTs by the same sponsors. For example, viewing conspirators as “alien lizards” seems so outlandish as to make other, more obviously minimally counter-intuitive CTs about corporate finance seem more intuitive. Identifying conspirators as alien lizards involves precisely the kind of not fully understood “quoted” mystery, as in specific religious dogmas such as the Trinity or Catholic transubstantiation. Moreover, the structure of apparently contradictory religious beliefs that in quotes is consistent with the finding that people simultaneously adhere to two contradictory CTs about the same focus (that Princess Diana was assassinated and that she staged her own death: Wood et al., 2012). Such a pattern suggest a “monological” conspiracy-related belief system that applies to more than one focus or topic (e.g., Swami et al., 2011). We suggest further that it has specifically quasi-religious qualities.

Often, however, agents are not viewed as maximally bizarre. They possess supernatural qualities of omniscience or omnipotence relative to the focus of the conspiracy, but not relative to other domains of knowledge or action – they are minimally counter-intuitive and minimally supernatural. For example, recurrent CTs regarding Jews have alleged their omniscience and omnipotence regarding medicine, astrology or alchemy in the Middle Ages (Zukier, 1987), or finance in the modern era. In such cases, the conspiring group is understood as all-knowing about the domain of action, but also as all-seeing regarding the sponsor’s knowledge about that domain. It is precisely this view of the conspirators as a strategic agent that supports the power of the CT. The agent group not only knows about the domain in question, but also knows what the sponsors know about the domain, and can therefore limit the sponsor group’s (or any other victim’s) scope for successful evasive action. Thus, adhering to such CTs abdicates self-agency. This strategic access is a recurrent theme in CTs. In the past, it might have been allied to supernatural ideas about the agents (e.g., Jews as in league with the devil, or as having magic powers; Zukier, 1987); in modern times it connects to the use of surveillance technology (e.g., as used by government agencies).

When referring to “supernatural” qualities, we do not mean that they are supernatural in the sense of being associated with non-existent beings; rather, we suggest that the CT represents the agents as having *minimally counter-intuitive strategically omniscient and omnipotent* qualities beyond the human norm. “Strategic” in this context implies that they are omnipotent and omniscience only relative to the particular domain that constitutes the focus of the conspiracy. Omnipotence supports their ability to carry out the conspiracy (they know about the domain so they can act to further their aims); omniscience allows them to be undiscovered or, if discovered, to remain unchallenged (they know what others (do not) know). It is this sense of supernatural that we intend, and not that agents are god-like beings, though sometimes (as in the Jewish CTs of the Middle Ages) such agents are recruited to the explanation. More mundane conspiracies instantiate the broader sense of the supernatural: for example, CTs regarding the assassination of John F. Kennedy allude to hidden groups who were able to carry out the murder and keep their role in it subsequently undiscovered.

***Proposition 1b: CTs are minimally counter-intuitive narratives.*** Actions of conspirators are framed in minimally counter-intuitive terms within minimally counter-intuitive narratives, which focus attention while remaining plausible explanations for threatening experiences. The narrative explains the conspiracy using accounts of everyday experiences and their connections to wider social conditions – everyday schemas, scripts, and stories that are used to explain other, non-conspiracy events are recruited in the CT explanation, to which they form the intuitive background. The CT propagators themselves can then offer minimally counter-intuitive narratives for awareness that promise redemption from the control of the conspirator. For example, the British conspiracy entrepreneur David Icke is “exposing the dream world we believe to be real” and exhorts people that it is “time to wake up” (). Icke’s narrative for action is that the wider population needs to become aware not only of the conspiracy against them, but also of their own hidden counter-intuitive agency that can challenge that conspiracy. The exaggerated agency of the conspirators can only be addressed by exaggerated agency of those conspired against. (Icke, 2013).

***Proposition 1c: CTs draw on culturally available images or essentialized inter-group differences in developing the minimally counter-intuitive narrative.*** Conspiracy theories draw on historical patterns of inter-group relations and representations. The role of essentialist characterizations may vary, but where CTs connect to historical inter-group hostility and prejudice, they inherit the essentialist structure of those inter-group relations – indeed, CTs may be the “ammunition” of those inter-group hostilities. Recurrent CTs depicting Jewish or Catholic agents exhibit such a structure, even as the imputed essences have changed (exactly as an essence placeholder would predict). The underlying qualities that enable counter-intuitive access and control have changed over time: where once they involved direct intercourse with a supernatural agent (the devil, for example) that conferred specific powers, now they involve exceptional intelligence, perhaps arising from genetic inheritance, plus cunning and access to technology and other resources. The structure of explaining conspiring activity by reference to underlying, group-based essences remains – despite changes in the alleged essences.

#### Proposition 2: Sharing CTs via communication rituals supports management of anxiety by transforming unspecific anxieties into focused fears

Communicating CTs is analogous to religious ritual – rehearsing answers to shared questions, as a declaration of faith and group identity (like the religious Creed). Indeed such rituals for CTs often take a reflexive form, providing not only answers to conspiracy-related questions, but also anticipating critique and denigration (e.g., Lewandowsky et al., 2013). Averting the denigration of the ingroup, possibly by outgroup denigration, is then a recurrent CT trope. Sharing core narrative cements sponsor ingroup identity and reinforces outgroup denigration; the general narrative of redemption from the control of the conspirators offers a focus for anxieties that threat generates. In this way, the vaguer threat arising from the alleged action and counter-intuitive powers of the conspiracy group is translated into focused fear and anger directed at that group. The transmission of CTs in this way overlaps with urban legends and rumors which also manage collective anxiety in the face of uncertainty (Kay et al., 2009). In the past, face-to-face communication in small communities would have provided fora for this; modern CTs have a plethora of technologies available for the same function. The internet, with blogs, chat rooms, and so on, is a fecund ground for CTs to flourish. This leads us to Proposition 3 with two components.

#### Proposition 3: CT narratives framed as conflicts over sacred values are more successful

***Proposition 3a: CTs framed as conflicts over sacred values are longer lived and less open to empirical rebuttal.*** Construing conspiring agents as a group committed to circumventing deeply held cultural beliefs is analogous to religious inter-group conflict over sacred values. As with the role of supernatural agents in CTs, we suggest an analogy with religious sacred values, not a homology. Many CTs concern the idea that a group is breaking the law for their own advantage. The very notion of an omnipotent, omniscient conspiracy group involves a denial of self-agency. This threat to agency may be experienced as a serious reason for outgroup derogation and conflict in cultures where the ideal of individual autonomy and freedom are cherished as “sacred” (e.g., Western individualistic cultures). In other cases, conflict may arise less over the alleged conspiracy *per se* than over the values and beliefs that are threatened by the conspiracy. In the case of corporations such as Monsanto, genetic modification is taken as a threat to the sanctity of life and reproduction, or as a threat to natural ways of eating. In still other cases, conflict may arise less over the domain of action for the conspiracy, but rather over the cultural origin of the alleged conspiracy group. This is exemplified by the Jewish conspiracies noted above. Yet another case would be the rejection by some Nigerian Muslim leaders of polio vaccines (Larson and Heymann, 2010) on the grounds that they form part of a Western conspiracy, in which the vaccines are alleged to threaten Muslim male fertility. In such cases, the conspiracy group is seen to act in contradiction to values that are deeply held – as sacred – by the sponsor group. A major outcome of this would be a lack of trust and a reduction of the likelihood of inter-group dialog and conciliation. Indeed, in conflicts over sacred values, attempts by one side to gain concessions by offering some benefit to the other (“buying them off”), may inflame the conflict and increase tensions (Atran and Ginges, 2012). An analogous pattern may arise in many ostensibly “secular” CTs: for example, believers in a CT in which “Big Pharma” meddle with Nature and human life could not be “bought off” by material concessions.

***Proposition 3b: CTs framed as conflicts over sacred values are more likely to lead to intense commitment and action.*** Whether a CT leads individuals to engage in collective action beyond an audience or a client “cult” to a “movement cult” via proselytism or fundamentalism depends on many factors. One is the interpretation of the conspiracy by adherents. If a conflict is understood as concerning “sacred” values (Ginges and Atran, 2009; Atran and Ginges, 2012), the CT provides a motivation and rationale for action against the conspirators. Another factor relates to the framing of other elements of the CT, the narrative, the agents and actors. CTs that identify specific actors who engage in specific actions at identifiable locations and times afford concrete counter-conspiratorial action. As Atran and Norenzayan (2004) has observed concerning the connection between “strong religion” and direct action (including terrorism), the causal drivers of action may concern personality differences, but more importantly depend on group, inter-group and cultural facilitation and direction. How believers interpret the threat and frame possible courses of inter-group action are key. It is to these matters that we now turn.

## EXPLAINING CT BELIEF AND SPREAD: THE THEORY OF SOCIAL REPRESENTATIONS

Cognitive science approaches to religion explain the appeal of CT beliefs by analyzing aspects of their content. CTs share many but not all such features of organized religion, which led us to characterize CTs as quasi-religious. Gervais and Henrich (2010) suggest that such models of religion are insufficient without an analysis of cultural milieus. The milieus or groups to which people belong predispose them to accept certain ideas more easily while rejecting others, as they make sense of the events to which they are exposed. These questions are addressed by social representations theory.

### SOCIAL REPRESENTATIONS AND “SPREAD”: COLLECTIVE SYMBOLIC COPING WITH SUDDEN SOCIAL CHANGE

Social representations theory (Moscovici, 1961; Bauer and Gaskell, 1999; Wagner and Hayes, 2005) investigates how laypersons make sense of novel and threatening situations in everyday life. Its premise is that modern societies are dominated by discourses provided by experts (particularly scientists) whose authority is not hegemonic (unlike Durkheim’s collective representations) but competes with other sources like tradition. The transition from collective representations in a hegemonic society to social representations in a context of plural mentalities may be a condition for the emergence of CTs in the first place. Roberts (1972) explains the historical emergence of CTs during the French revolution as a desperate attempt to retain a collective representation of society in the image of God and interpreted by the Church. Under a religious monopoly of interpretation, the revolution could only have been the work of evil and its avatars (see the influential work of Father Augustin de Barruel on the French Revolution as a Jacobin-Freemason conspiracy in Roberts, 1972). Modern pluralism and pragmatism might be a historical condition of both recognition and proliferation of CTs.

Expert discourses (corresponding to domains of scientific knowledge) are popularized and circulate via the mass media where they are apprehended by laypersons who use them to make individual and collective decisions. Examples include health-related issues (e.g., dieting, cancer screening), consumer decision-making (e.g., sustainability, fair trade, and ethical purchasing) or the societal implications of new technologies (nuclear energy in the 1970s and 1980s, biotechnology in the 1990s; Bauer, 1995). Such situations confront laypersons with unfamiliar knowledge, of which they must develop a working understanding. Beck (1992) and Giddens (1990) have characterized this condition as the “risk society”: the negative repercussions of modern technologies are uncontainable within national boundaries. Extensive research in economics and psychology has highlighted the differences between expert and lay understandings of risk (Slovic et al., 1982; Tversky and Kahneman, 1974). And communication scientists have studied how to best convey information about risk to the public, and how to train the public to make proper use of it (Fischhoff, 1995). Much of this research is based on the “deficit model” of public understanding of science (Wynne, 1991; Ziman, 1991) according to which laypersons lack information or use biased cognition in their decision-making.

The social representations approach investigates similar phenomena, but rather than highlighting the deficits of public understanding, it focuses on the processes by which laypersons reconstruct expert knowledge to enable social and pragmatic functioning in everyday life. These processes translate the unfamiliar, abstract and often threatening aspects of expert knowledge domains into familiar and mundane terms. They involve symbolic resources from popular culture, religion, or group-specific ideologies. In this respect, a social representation is a hybrid creation fusing popularized science knowledge and other forms of cultural knowledge (Wagner, 2007). The function of a social representation, by making the unfamiliar familiar (Moscovici, 1984), is to protect the symbolic integrity of a group’s worldview, a worldview often threatened by expert discourse. Social representations thus frame unfamiliar events in more familiar terms. (1974, p. 22) distinguished between two basic interpretive frames. Natural frames “identify occurrences as seen as undirected, unoriented, unanimated, unguided, “purely physical”. Social frames “provide background understanding for events that incorporate the will, aim and controlling effort of an intelligence, a live agency.” In these terms, elaboration of social representations often entails replacing a natural framing of events with a social framing.

There are two processes by which social representations are elaborated: Anchoring and objectification. Anchoring is naming an unfamiliar object or event. By naming, an object is classified and positioned in a familiar semantic field which links it to other things which it normally goes with or can be substituted by, and makes it distinct from opposites. Anchoring an event makes it recognizable and less threatening, not least because it can be discussed by members of the group. In Moscovici’s (1961) study of the popularization of psychoanalysis, the abstract domain of psychoanalysis was assimilated to the confession in the Catholic press. This allowed Catholic laypersons to anchor a key feature of psychoanalysis within their worldview and make it less threatening. Objectification is another way of dealing with the unfamiliar, whereby abstract contents are made tangible through a metaphor or a concrete visual image. Examples of objectification include the everyday image of the atom as a ball (Wagner and Hayes, 2005), the image of the couch as a symbol of psychoanalysis (Moscovici, 1961), or the tendency to portray microorganisms or genes as intentional agents (Wagner et al., 1995; Bangerter, 2000; Green and Clémence, 2008). In the terms outlined above, anchoring and objectification transform the threatening and maximally counter-intuitive into the surprising but minimally counter-intuitive. For example, the psychoanalyst is seen as having the intuitive properties of a priest but with the minimally counter-intuitive property of dealing with the unconscious rather than the soul.

Anchoring and objectification are two facets of the reconstruction of expert knowledge in common sense, a communicative process that is played out in the mass media, but also in conversations between individuals (Moscovici, 1984). Similar to rumor, this communication process is driven by the need to understand and make sense of the unfamiliar object or event (Green and Clémence, 2008). Indeed, the intensity with which a rumor spreads is a joint function of an event’s importance and its ambiguity (Allport and Postman, 1947; Rosnow, 1980; Franks and Attia, 2011).

Social representations theory has an additional facet that makes it well-equipped to deal with lay thinking, especially relative to CTs. Often, laypersons are confronted with unfamiliar techno-scientific domains in an abrupt manner, as events emerge that require attention, interpretation, and action. The elaboration of a relevant social representation serves a process of collective symbolic coping by which groups manage the implications of events that propel social change (Wagner et al., 2002). Collective symbolic coping is analogous to the process of individual coping in stress research (Lazarus and Folkman, 1984). Wagner et al. (2002) proposed that collective symbolic coping proceeds according to four stages: awareness, divergence, convergence, and normalization. Awareness is when an event is made into an issue relevant for the public by the media. Divergence is when public discourse about the issue leads to the emergence of multiple frames or interpretations, creating a symbolic environment characterized by ambiguity or uncertainty. Convergence is when a dominant frame emerges, suppressing competing frames and decreasing uncertainty. Finally, normalization corresponds to an incorporation of the event into everyday knowledge. The event ceases to be threatening.

Wagner et al. (2002) illustrated collective symbolic coping with the emergence of biotechnology in Europe during the 1990s. During the coping phases, the prevalence of beliefs in threatening or fantastic images of biotechnology was higher than before or after coping. Another example comes from the emergence of new infectious diseases (e.g., Ebola virus, SARS, avian influenza, and most recently, the 2009 H1N1 influenza, or “swine flu” pandemic). Expert discourses on disease risk have been propagated widely in the media. But laypersons have difficulty interpreting these discourses (framed in the abstract, probabilistic terms of risk science). Collective symbolic coping with disease often entails the elaboration of representations identifying a culprit, typically a group responsible for the outbreak (Joffe, 1999).

### APPLICATION TO CTs

In general, then, social representations constitute normatively incorrect but functional knowledge structures elaborated by laypersons on contact with expert knowledge. Social representations help groups to symbolically cope with a new or threatening event, by making abstract risk concrete, often by focusing blame. They thus contrast with expert knowledge, developing a subversive character. These properties make the theory of social representations useful for understanding CTs: as above, we express this in a series of empirically testable propositions.

#### Proposition 4: the spread of CTs operates via the processes of anchoring and objectification

An approach to CT spread using social representations has been proposed by Byford (2002), who notes that CTs evidence a “fluid and dynamic quality” (Byford, 2002, p. 3.3), i.e., a wide variety of forms, while at the same time sharing some characteristics. In trying to pinpoint key structural features of CT narratives, researchers have classified the agent groups to which the conspiracy is attributed, with some suggesting CTs stigmatize minorities (Moscovici, 1987), and others that they frame events as the work of “evil elites” (Campion-Vincent, 2005). Both kinds of CT exist and their appeal depends on specific individual differences like ideological preferences, for example (Wagner-Egger and Bangerter, 2007). Minority CTs identify stigmatized outgroups as scapegoats for events, for example blaming Jews for the plague, Muslims for plotting against Western societies, or Communists for plotting to overthrow democracy. Evil elite CTs tend to focus on powerful organizations that orchestrate events behind the scenes in a bid for world domination, such as the Bilderberg Group. Both types constitute general frames within which current events can be flexibly accommodated in a conspiratorial worldview, even to the point of glossing over contradictions between alternative CTs (Wood et al., 2012). This corresponds to the anchoring process by which complex, abstract expert-dominated discourses about causes of events get reinterpreted as a personified conspiracy. Telling examples of objectification are abundant in the CT genre, especially since their mass media diffusion involves a strong visual component. Recurrent images associated with CTs include the all-seeing eye of the Illuminati as an embodiment of omniscience, or depictions of reptilian aliens or lizards at the heart of many CTs, similar to the figure of the devil, the original conspirator (Jacques-Chapin, 1987).

#### Proposition 5: CTs are devices to cope with collective trauma via their development and spread as social representations

Traumatic collective events need to be assimilated by the groups that experience them (Pennebaker et al., 1997). This often involves constructing a cause, especially for seemingly random events, like the car accident in which Princess Diana was killed. Ascribing causality allows coping with the loss of control caused by the experience (Kay et al., 2009), according to the maxim that big events (e.g., the death of a celebrity) call for big causes (a conspiracy; McCauley and Jacques, 1979; Leman and Cinnirella, 2007). The symbolic elaboration of a powerful, shadowy conspiring group as cause of the event is a way to do justice to its emotional importance.

#### Proposition 6: CTs’ rejection of authoritative discourses arises from how expert knowledge gets reconstructed as a social representation

Recall how collective symbolic coping progresses through stages of awareness, divergence, convergence, and normalization. In these terms, CT spread corresponds to a “denormalization” of a dominant framing of an event: an account that problematizes that frame, construing it as a sham and suggesting alternative truths. Consider the example of AIDS, which has been in a process of normalization since the 1990s (Rosenbrock et al., 2000): its image has transformed from a deadly plague to a serious, but chronic and manageable disease, and there is a consensus that AIDS is caused by the HIV virus. CTs about AIDS (e.g., propagated by AIDS denialist movements, Kalichman, 2009), reject this cause, suggesting that AIDS is caused by environmental or lifestyle factors. Militant action is aimed at undermining the dominant framing of AIDS.

Another aspect of this concerns the “choice” of outgroup, which may depend both on the milieu of sense-making and on specific individual differences and ideological preferences; right-wing politics may typically mobilize against minorities, whilst left-wing politics incriminate powerful elites (Wagner-Egger and Bangerter, 2007). This also suggests that CTs are a likely risk of extremist politics (Inglehart, 1987; Hogg and Blaylock, 2011).

## EXPLAINING HOW CTs MOBILIZE COLLECTIVE ACTION: FRAME THEORY

Many CTs constitute means of making sense of complex events by ascribing them a clear cause, often focusing blame on a culprit. The psychological and socio-cultural qualities of CTs can mobilize resistance to powerful organizations, such as multinational corporations or political tyranny. Alternatively, CTs may generate a highly cynical form of inter-group engagement in civic society, reducing trust, and driving strategic action which undermines the functioning of a modern public sphere (in the sense of Habermas, 1981). This is where much of the public concern about modern CTs arises, since they can create a climate of mistrust or lead to disengagement from mainstream society or from officially recommended practices. In the US, Blacks who believe that AIDS is a White conspiracy against Blacks are less likely to practice contraception, probably because of mistrust toward the public health system (Thorburn and Bogart, 2005). In other cases, CTs may be more widely believed and contribute to social and political engagement (e.g., Sapountzis and Condor, 2013).

But not all CTs lead to collective mobilization. Activist groups like AIDS denialists actively propagate beliefs about the scientific establishment attempting to hide the truth about AIDS. But examples like these are rare. There are no sustainable social movements dedicated to revealing the truth about Princess Diana’s demise, for example, or to making the purportedly faked moon landings a high-profile public issue. Why do some CTs mobilize groups into action while many others do not? Social representations theory does not really focus on CTs’ potential for mobilization (Klein and Licata, 2003); mobilization requires a blueprint for action in addition to sense-making. Frame theory as applied to social movements has specified the constitution of such blueprints, in a way compatible with social representations theory.

### FRAME THEORY AND COLLECTIVE ACTION

A frame is a mechanism of selection, of inclusion and exclusion of elements that shape our understanding of experience. Goffman (1974, p. 21) called them “schemata of interpretation,” and, as described above, distinguished two basic frames: natural and social. In the social movement literature, framing is seen as an alternative to structural explanations of collective action. Collective action does not follow automatically from ideological bent, but rather because individuals are emotionally aroused by issues and attached to symbols worthy of their loyalty. Framing is the process of construction of these issues or symbols by a social actor, who has to gain from mobilization around the issue. Framing moves groups into action, recruiting bystanders, while demotivating and demobilizing antagonists (Tarrow,1994; Benford and Snow, 2000).

Mass media frames name and solve a problem by providing structures of cognition and interpretation: “To frame is to select some aspects of a perceived reality and make them more salient in a communicating text, in such a way as to promote a particular problem definition, causal interpretation, moral evaluation, and/or treatment recommendation for the item described” (Entman, 1993, p. 52). Frame elements are enhanced by a key metaphor and eye-catching visualizations (Gamson and Modigliani, 1989). Importantly, framing collective action goes beyond interpretation to cover three steps: diagnosis, prognosis, and motivation (Benford and Snow, 2000). Diagnosis involves identifying a problem and attributing it to a cause, i.e., focusing blame on a target. Prognosis involves “articulation of a proposed solution to the problem, or at least a plan of attack, and the strategies for carrying out the plan” (Benford and Snow, 2000, p. 616). Motivation involves constructing a rationale for action, for example by elaborating discourses describing the severity or urgency of the issue framed. Social representations theory and frame theory both focus on diagnosis. However, social representations theory is less specific on prognosis and motivation.

Framing for strategic action via prognosis and motivation may initially be a shot in the dark where the conditions for successful action are not clear. The effectiveness of a frame in garnering support is its resonance (Benford and Snow, 2000). Frame resonance is a function of its credibility and its salience. Credibility is itself a function of the consistency of the frame, its empirical credibility, and the credibility of the actors promoting the frame. Salience is the degree to which the frame elements are important to the intended targets. Moreover, a frame needs to cross the divide between specialist channels and the mass media to succeed in mobilization (Strodthoff et al., 1985).

Frames also have a temporal dimension in a culture; they come and go. This is captured by research on *issue-attention cycles*. Starting from the notion that the polity does not react to grievance *per se*, but rather to mass mediation of grievance, Downs (1972) suggested that news ebbs and flows in five phases constituting an issue-attention cycle: in the initial pre-problem stage, grievances receive little public attention. Some dramatic event, e.g., an accident, triggers an alarmed discovery of grievance. The public takes notice and develops a pressing sense that “something needs to be done.” During a third phase, the costs of solving the problem become clear, and the public becomes aware that the grievance was beneficial for some. After action, public attention is likely to wane into a gradual decline (fourth phase); the news space is taken over by other novel issues. Finally, post-problem the issue moves into an unstable limbo. Some actor might monitor the issue and keep its memory, so it can flare up sporadically and be kept from oblivion. Such issues cycles map the course of mass media attention in a public arena where capacity is limited, e.g., as when other issues compete for attention (Hilgartner and Bosk, 1988).

### APPLICATION TO CTs

By their nature, all CTs function as diagnostic frames. However, not all establish a prognosis (beyond vague calls to “wake up”) and very few elaborate discourses calling for specific action with regard to identified culprits. And to the extent that a given CT frames its adherents as lacking relevant agency (and the conspirators as strategically omnipotent), it is likely to engender precisely the kind of social and political disengagement noted above. Our propositions below should be seen in this context.

#### Proposition 7: CTs can generate collective action when their frames are credible and salient

Some CTs motivate coordinated collective action for short periods of time, often using channels of communication and action that are outside the norm. This is often the case for fringe political movements (Inglehart, 1987). The Occupy movements of 2011–2012 were successful in mobilizing action for a short time because of an intuitive diagnosis (“Wall Street” as a symbol of evil finance and the culprit for the latest financial crisis), prognosis, and motivation, by specifying a concrete course of action (“occupy”), and by a claim of salience and credibility relevant to many constituencies of the public, which connected the action to deeply held values of democracy and fairness (“We are the 99 percent”). At the other end of the political spectrum, the right-wing US Tea Party attracts anxiety-driven adherents of CTs about government (Barreto et al., 2011).

What makes a CT frame credible or salient? As noted above regarding the quasi-religious content of CT beliefs, the change from a client- or audience-cult approach to a CT, to involvement in a cult-movement and onto further committed practical action seems to involve framing with specific contents, targets or locations for counter-conspiracy action. A specific driver for action may also be the extent to which a conspiracy is framed as contradicting sacred values.

The success of such framing may depend on the credibility of those who frame communications to the group or who lead the group (e.g., Turner and Killian, 1957; Reicher, 1987). If opinion leaders regarding the CT are credible, their framing of the conspiracy as a normative clash over sacred or deeply held values may be an important spur to action. Framing also offers a way of interpreting the findings of Sapountzis and Condor (2013) regarding CTs in Greek citizens’ discussions of the Macedonian crisis. They found that CTs echoed past and future threats to Greece (thus establishing credibility via tradition and future projects) and also transmuted the understanding of inter-group threats from being concerned with symbolic competition to include realistic competition (establishing salience and relevance). Mobilizing a CT to challenge the established inter-group frame of reference – to denormalize that frame, as in Proposition 6 above – provides fertile conditions for collective political action. Indeed, some CTs may motivate action by taking a relatively vague, existential anxiety, translating it into a symbolic inter-group threat, and then transmuting this into a more specific realistic conflict over resources – where the latter gives tangible grounds and focus for action.

However, many CTs fail to engender action. Some that are widely held, like JFK assassination CTs, fail to relate the diagnosis to a prognosis and motivation – perhaps because no obvious action is relevant. Other CTs may fail to mobilize collective action because they lack sufficient resonance – credibility or salience. For example, that white US college students are less likely to believe in CTs depicting the US government as conspiring against Blacks than Black students are (Crocker et al., 1999) may reflect insufficient frame salience.

#### Proposition 8: CTs fluctuate in their cultural success as a result of issue-attention cycles

Issue-attention cycles illustrate some of the difficulties evidenced by CTs in eliciting sustained mainstream attention. Consider the so-called “satanism ritual abuse movement.” Various interest groups in the US in the 1980s (religious groups, therapists, politicians, and other public officials) and mass media propagated stories of satanic cult activity. Allegedly, such cults were engaging in conspiratorial activities designed to undermine Christianity and civil society, including kidnapping and blood sacrifice rituals (Richardson et al., 1991). Rumors of cult activities captured the attention of the US public with a moderate degree of intensity for several years. The mainstream success of this CT can be explained by several factors from frame theory; the spillover into the mass media, the construal of Satanism as undermining sacred values of contemporary US society, and the enrollment of credible specialists to convey such ideas. However, such a configuration of interested parties was not sustainable in the absence of real evidence and attention waned in the 1990s.

## CONCLUSION

Conspiracy theories are a feature of common sense. We have suggested that they involve quasi-religious explanations of threatening events. In that sense CTs express the coexistence of “older” and ”newer” mentalities. The quasi-religious hypothesis aims to elucidate the dynamics of CTs and thereby address the three key questions that arise regarding the distribution of representations and their connection to social movements: the questions of “stick,” “spread,” and “action.”

### THE QUASI-RELIGIOUS HYPOTHESIS

Our main hypothesis regarding what makes CTs “stick” to explain threats, is that they incorporate quasi-religious, minimally counter-intuitive representations of external agents who are omniscient and omnipotent regarding the domain of that threat. Ascribing supernormal agency to the conspirators connects the conspiracy to historical inter-group relations and conflicts, so that the social groups involved are also represented in hard-to-falsify essentialist terms, which naturalize the differences and explanation. These elements of religious thinking are recruited for a flexible, pragmatic, and secular explanation. Such representations of conspirators also contribute to the psychological anxiety managing function of CTs: translating diffuse anxiety about a threat into specific, historically recurrent fears and inter-group dynamics.

The main hypothesis regarding CT’s spread is that social representations anchor threatening events in terms of already familiar understandings, and objectify them in concrete terms. CTs are rooted in long-established contents and patterns of thinking. This suggests that widespread CTs should typically involve easily identified outgroups, either in stigmatized minorities or powerful elites whose agency is implicated and exaggerated.

To the extent that CTs frame events credibly and specifically enough to direct action, and in strong evaluative terms (e.g., regarding conflicts over sacred values), they may lead to counter-conspiratorial social action. However, the way CTs bracket the agency of the ingroup and emphasize the agency of the all-powerful outgroup can foster disempowerment, inaction on the part of CT believers or a call to opt out of society. Transmuting inaction into counter-conspiracy action therefore requires quite specific additional cultural and inter-group influences to be brought to bear. These involve empirical credibility and social prestige of CT sponsors, and opinion formers.

### THE QUASI-RELIGIOUS HYPOTHESIS AND OTHER APPROACHES

Insofar as our account emphasizes overinterpreted agency as a means of managing collective anxiety, the quasi-religious hypothesis overlaps with other accounts of CTs. Other accounts argue for the centrality of ideology, discourse or political beliefs in CTs’ anxiety management (e.g., Hogg and Blaylock, 2011; Van Prooijen, 2011), or for agency issues *per se *in CTs (e.g., Shermer, 2012; Swami and Furnham, 2012), without adverting to religion. Such accounts are powerful and illuminating.

However, our suggestion is that analogies between CTs and religion have specific implications beyond those from ideology or from agency. One such implication is the intrinsic indeterminacy in quasi-religious framings (the kernel of doubt in the structure of quoted beliefs). We suggest that CTs draw on beliefs that are not fully understood or interpreted, where the details of the conspiracy are not usually fully spelled out. Other views stress the role of CTs in responding to uncertainty; we suggest that the quasi-religious response itself incorporates uncertainty and indeterminacy. Such indeterminacy underpins the quasi-religious view, and supports the 13 specific propositions that we have articulated. Other empirical consequences follow from the hypothesis – for example, that the indeterminacy generated by the kernel of doubt generates resistance to counter-examples and falsification; or that established CTs offer scope for schism and intra-group division as do religious beliefs. There is similar specificity to the empirical implications of the ways in which social representations theory addresses sense-making, via anchoring and objectification; and to the ways in which framing connects to collective action.

Put another way, we concur with accounts explaining CTs relative to ideology and culture or agency, but suggest that CTs have an array of specific quasi-religious qualities that are complementary to, and instantiate, those accounts in particular ways that draw on the dynamics of social representations and frame theory. We suggest that the religious analogy can be a fertile, while more constrained, empirical approach.

### CTs, QUASI-RELIGIOUS BELIEFS, AND CIVIL SOCIETY

The elements of stick, spread, and action, whilst analytically separable, are not separate sequences of CTs. Indeed, their overlap leads to the prediction that CT spread is not simply a repetition and sharing of a “finished” representation; rather, as the CT spreads it is elaborated and embedded into social representations that may frame collective action. A particular path of this is the progressive normalization of the CT, involving the essentialization of outgroups and their minimally counter-intuitive capacities. Once a CT is normalized it becomes an incontrovertible, taken-for-granted part of commonsense. The CT narrative can then function as an interpretive template for other threats. Conspiratorial thinking is therefore mutable and portable across threats and domains.

The quasi-religious account thus offers a way of interpreting the paradoxical relations between CTs and modern civil society. On the one hand, cultural conditions of pluralism and secularism are preconditions for the “bricolage” that engenders a CT. But on the other hand, specific CTs may attempt to counter such pluralism, by denigrating outgroups that might have power similar to or greater than the ingroup (for example in recurrent anti-Jewish CTs). Moreover, CTs offer an “opportunity” for civil society, in supporting the emergence of issue-based social movements that underpin much social change; in this sense CTs can challenge established economic and political disparities. But, CTs also threaten civil society, since such quasi-religious ways of thinking engenders uncompromising fundamentalism that decrease the prospects of fruitful inter-group dialog. CTs undermine civil society when they mobilize for exit rather than voice, or by framing violent actions against identified targets, be they powerful actors or stigmatized minorities. Hence, as religious beliefs, CTs do not necessarily threaten civil society.

Our proposal has implications for future research. Investigating our propositions means exploring the cultural, social, and cognitive psychological processes that connect “stick,” “spread,” and “action.” In this way, we can gauge how a CT becomes a threat or an opportunity for civil society. Determining how and why specific CTs become threats whilst others offer opportunities will require a case-by-case comparative approach which integrates individual, social, and cultural levels of analysis, and examines the specific contents of a CT, its inter-group milieu, and its cultural transmission processes and contexts. It would be too risky to dismiss *a priori* the dynamics involved in CTs as socially dysfunctional. In this paper we have rather assumed that dysfunction is a secondary development of what is primarily a functional feature of social life, i.e., the commonsense attribution of agency to explain threats and dangerous events.

## Conflict of Interest Statement

The authors declare that the research was conducted in the absence of any commercial or financial relationships that could be construed as a potential conflict of interest.
